# Application of compost amended with biochar on the distribution of antibiotic resistance genes in a soil–cucumber system—from the perspective of high-dose fertilization

**DOI:** 10.3389/fmicb.2025.1530296

**Published:** 2025-03-10

**Authors:** Shuai Shi, Zhenye Tong, Bo Sun, Yiyang Wei, Yu Tian, Qihui Zuo, Xingxing Qiao, Jiaze Duan, Wenlong Bi, Junmei Qin, Jun Zhou, Fenwu Liu

**Affiliations:** ^1^College of Resources and Environment, Shanxi Agricultural University, Jinzhong, China; ^2^Shanxi Dadi Environment Investment Holdings Co., Ltd., Taiyuan, China; ^3^Research Institute, College of Biotechnology and Pharmaceutical Engineering, Nanjing Tech University, Nanjing, Jiangsu, China; ^4^Nongshengyuan Family Farm, Jinzhong, China; ^5^Key Laboratory of Sustainable Dryland Agriculture (Co-construction by Ministry and Province), Ministry of Agriculture and Rural Affairs, Shanxi Agricultural University, Jinzhong, China

**Keywords:** compost, antibiotic resistance genes, bacterial communities, mobile genetic elements, soil–cucumber system

## Abstract

The transfer of antibiotic resistance genes (ARGs) from soils to vegetables negatively impacts human health. This study explored the effects of the high-dose (18.73 t/ha) application of traditional compost (TC) and composts produced through the co-composting of traditional materials with large-sized (5–10 mm) biochar-amended compost (LBTC) or small-sized (< 0.074 mm) biochar-amended compost (SBTC) on the distribution of ARGs in a soil–cucumber system were explored. Results indicated that the SBTC group had the highest soil nitrogen, phosphorus, and potassium contents, followed by the LBTC, TC, and control treatment groups. These findings aligned with the quality and weight of harvested cucumbers. Bacterial community diversity decreased in compost-fertilized soils. Compared with their preexperimental values in soils, the total absolute abundances of ARGs and mobile genetic elements (MGEs) increased by 23.88 and 6.66 times, respectively, in the control treatment group; by 5.59 and 5.23 times, respectively, in the TC group; by 5.50 and 1.81 times, respectively, in the LBTC group; and by 5.49 and 0.47 times, respectively, in the SBTC group. Compared with those in the control treatment group, the absolute abundance of *ermB*, *ermT*, *gyrA*, *qnrS*, *tetC*, and *intI1* decreased by 6–100% in the soil of the SBTC group. Compost application to soils significantly decreased ARG abundance in cucumbers; SBTC had the most significant effect and reduced the number of host bacteria at the phylum level from four to three. Nutrient levels in soils were important factors influencing the migration of ARGs from soils to cucumbers. In summary, when compared to other composts, the high-dose (18.73 t/ha) application of SBTC is more effective at reducing the risk of the accumulation and transfer of ARGs in the soil–cucumber system.

## 1 Introduction

Antibiotic resistance genes (ARGs), a new contaminant, pose serious risks to human life and health through their spread among environments (Hernando-Amado et al., 2019). The number of annual deaths from treatment failure due to antibiotic resistance is approximately 700,000 and will reach 10 million in 2050 (Shao et al., 2022). The frequent use of antibiotics in livestock farming and their low uptake rate by animals result in livestock manure being the main reservoir of antibiotics and ARGs (Gao et al., 2024). The application of livestock manure as an organic fertilizer on agricultural soils enhances the formation of the transmission chain of ARGs from manure to soil and subsequently to crops (Wang et al., 2022). Although livestock manure is rich in nutrients and organic matter and promotes plant growth, it accelerates the spread and accumulation of ARGs in soil–plant system (Zhang et al., 2019). It also results in the transfer of ARGs into the human body through agricultural crops and vegetables. Moreover, a previous study found that the abundance of ARGs in organic vegetables was 9 times greater than that in regular vegetables (Zhu et al., 2017). However, studies on reducing the spread of manure-derived ARGs in soil–vegetable systems are scarce.

Composting is an effective method for removing ARGs from manure (Awasthi et al., 2019). Compared to the direct application of manure, manure compost reduces the risk of spreading ARGs in soils (Gou et al., 2018). In soils, mobile genetic elements (MGEs) and microbial communities play key roles in the proliferation and spread of ARGs, which depend on propagation of host bacteria and horizontal gene transfer through MGEs (Zhu et al., 2022). The accumulation of MGEs results in the frequent acquisition of exogenous ARGs by host bacteria through horizontal gene transfer (Groussin et al., 2021). This phenomenon further increases the multidrug resistance of ARGs and complicates their removal from soils. A previous study found that host bacteria can transfer ARGs and MGEs from soils to plants (Wang et al., 2022), thus posing a threat to the safe production of vegetables. However, traditional manure composting demonstrates low removal efficiency for ARGs and MGEs (Riaz et al., 2020).

Research shows that the addition of biochar can enhance the removal of MGEs and ARGs during manure composting, and reducing the size of biochar size demonstrates a remarkable effect (Tong et al., 2023b). Biochar has a large specific surface area, a rich porous structure, and surface functional groups, which help inhibit the proliferation and diffusion of ARGs through absorption and decomposition; these characteristics also influence bacterial communities and horizontal gene transfer via MGEs, leading to a decrease in the level of ARGs (Fu et al., 2021; Fang et al., 2022). Composted biochar has high porosity, numerous pore structures, and small surface pores (Hagemann et al., 2017), which may further enhance its inhibitory effect on the proliferation of ARGs in soils. The decrease of ARGs in soils lays a strong basis for decreasing the accumulation of ARGs in vegetables. However, the 4.68 t/ha application of compost amended with biochar with different sizes increases the abundance of ARGs in soils and only inhibits the spread of *sul1* and *tetG* from soils to cucumbers (Tong et al., 2023a). This may be due to the minor effect of low application amounts of compost on the decrease in ARG levels in soils and cucumbers. A previous study found that applying manure composts at a dose of 30 t/ha significantly decreased ARG levels in soils (Sun et al., 2023). The compost application led to a transient increase in ARGs in the soil, which decreased to below-background levels after 4 months (Xu et al., 2019). Therefore, further in-depth exploration of the effects of high-dose soil application of biochar-amended composts with varying sizes on the proliferation and spread of ARGs in soil–cucumber systems is worthwhile.

The aims of this study are to (1) explore whether the high-dose (18.73 t/ha) application of biochar-amended composts with various sizes decreases the levels of ARGs in a soil–cucumber system; (2) evaluate the impact of the high-dose (18.73 t/ha) application of composts on cucumber quality and soil bacterial communities; and (3) identify the primary factors affecting the distribution of ARGs in the soil–cucumber system.

## 2 Materials and methods

### 2.1 Experimental design

This experiment was conducted as a field trial, and the relevant experimental site is located in Shagou Village, Taigu District, Jinzhong City, Shanxi Province, China (37°27′35.55′′N, 112°38′8.002′′E). The study included four groups in its design. They were Group CK without applied composts to soils, Group with applied traditional compost (TC: a mixture of pig manure and corn straw co-composted for 50 days) to soils, Group with applied large size (5–10 mm) biochar-amended compost (LBTC: a mixture of pig manure, corn straw, and large size biochar co-composted for 50 days) to soils, and Group with applied small size (<0.074 mm) biochar-amended compost (SBTC: a mixture of pig manure, corn straw, and small size biochar co-composted for 50 days) to soils.

TC, LBTC, and SBTC were produced in an earlier composting experiment (Tong et al., 2023b); their physicochemical properties were described in a previous study (Tong et al., 2023a). Three replicates were set up for each group; each replicate had a plot with a length of 2 m and a width of 3 m. A total of 11.24 kg of the relevant compost was applied to the soil of each plot (i.e., 18.73 t compost/hm^2^). Urea (*N* ≥ 46%), calcium superphosphate (P_2_O_5_ ≥ 12%), and potassium sulfate (K_2_O ≥ 60%) were applied to maintain nitrogen, phosphorus, and potassium at the same level in soils, and their usage in each plot of different fertilization groups is shown in Supplementary Table S1. The cucumber variety utilized in this study was consistent with that used in a previous study (Tong et al., 2023a). The physicochemical properties and total absolute abundance of ARGs and MGEs in the soils in different groups prior to this experiment were aligned with those of the soils after applying the same composts in a previous study (Tong et al., 2023a), as shown in Supplementary Table S2.

### 2.2 Collection of samples and measurement of relevant indicators

The soil samples of different groups were collected from 0–20 cm below the soil surface when cucumbers first matured. Five cucumbers from the first harvest in each plot were selected for quality measurement. The total soluble sugar, soluble protein, vitamin C, nitrate, and nitrite contents in cucumbers were measured using Ferrin’s reagent method, Kaunas’ brilliant blue G-250 staining method, 2,6-dichlorophenol indophenol titration, UV spectrophotometry, and the naphthalene ethylenediamine hydrochloride method, respectively. The details of these determinations are shown in the Supplementary material.

Fresh soil samples were used to measure moisture content and nitrogen in ammonium, nitrate, and nitrite forms. Air-dried soil samples were used to detect organic matter, total nitrogen, total phosphorus, total potassium, available phosphorus, available potassium, available nitrogen, pH, and electrical conductivity (EC). Cucumber yields were measured as the total weights of cucumbers from the 10 collections. The methods used to measure the aforementioned soil physicochemical properties were aligned with standard methods, and their details are shown in the Supplementary material. Fresh samples collected as previously described were stored in an ultralow temperature freezer at −80°C for ARGs measurements and 16S ribosomal RNA (rRNA) sequencing.

### 2.3 DNA extraction and qPCR quantification of antibiotic resistance genes and mobile genetic elements

A genomic DNA extraction kit was used to isolate DNA from the soil and cucumber samples following the manufacturer’s instructions. Genomic DNA extraction was replicated 3 times for each sample. The quality and concentration of the extracted DNA were assessed using 1% agarose gel and NanoDrop 2000c (Thermo Scientific, Fargo ND, USA). Subsequently, two MGEs (*intI1* and *intI2*) and 20 ARGs (*mefA*, *mphA*, *gyrA*, *qnrS*, *ermB*, *ermC*, *ermF*, *ermT*, *sul1*, *sul2*, *tetA*, *tetB*, *tetC*, *tetG*, *tetM*, *tetO*, *tetQ*, *tetW*, *tetX*, and *tetZ*) were quantified by using an RT-qPCR 7,500 system (Thermo Scientific, Fargo ND, USA). The relevant primer sequences for the amplification of ARGs and MGEs are shown in Supplementary Table S3. The thermocycling conditions for amplifying ARGs and MGEs in this study were consistent with those used in a previous study (Tong et al., 2022). The absolute abundance of ARGs and MGEs was calculated as follows: copies/g = (copy number × DNA volume × dilution times)/sample dry weight.

### 2.4 Soil bacterial community analysis based on high-throughput sequencing

Successfully extracted DNA from soil samples was further used for high-throughput sequencing to reveal bacterial communities in soils. The primers 341F and 806R, which target the V3–V4 region of 16S rRNA, were used to analyze soil bacterial communities. Small-fragment library construction and paired-end sequencing based on the Illumina NovaSeq-PE250 sequencing platform Illumina, USA (MiSeq-PE300, Illumina, San Diego, CA, USA) were performed with the help of Wcgene (Shanghai, China).

### 2.5 Data analysis

Data were statistically analyzed in triplicate with Statistical Package for the Social Sciences (SPSS) version 25.0 (IBM, Armonk, NY, USA). Differences in the soil properties and cucumber qualities of different groups were analyzed by using a one-way analysis of variance. TBtools was applied to generate the abundance heat map of bacteria at the genus level in soils and ARG abundance in cucumbers. The network analysis based on Person’s analysis and the establishment of a structural equation model (SEM) was performed using Cytoscape 3.9.0 and Amos Graphics (IBM, Armonk, NY, USA).

## 3 Results and discussion

### 3.1 Changes in soil physicochemical properties

**Figure 1:**
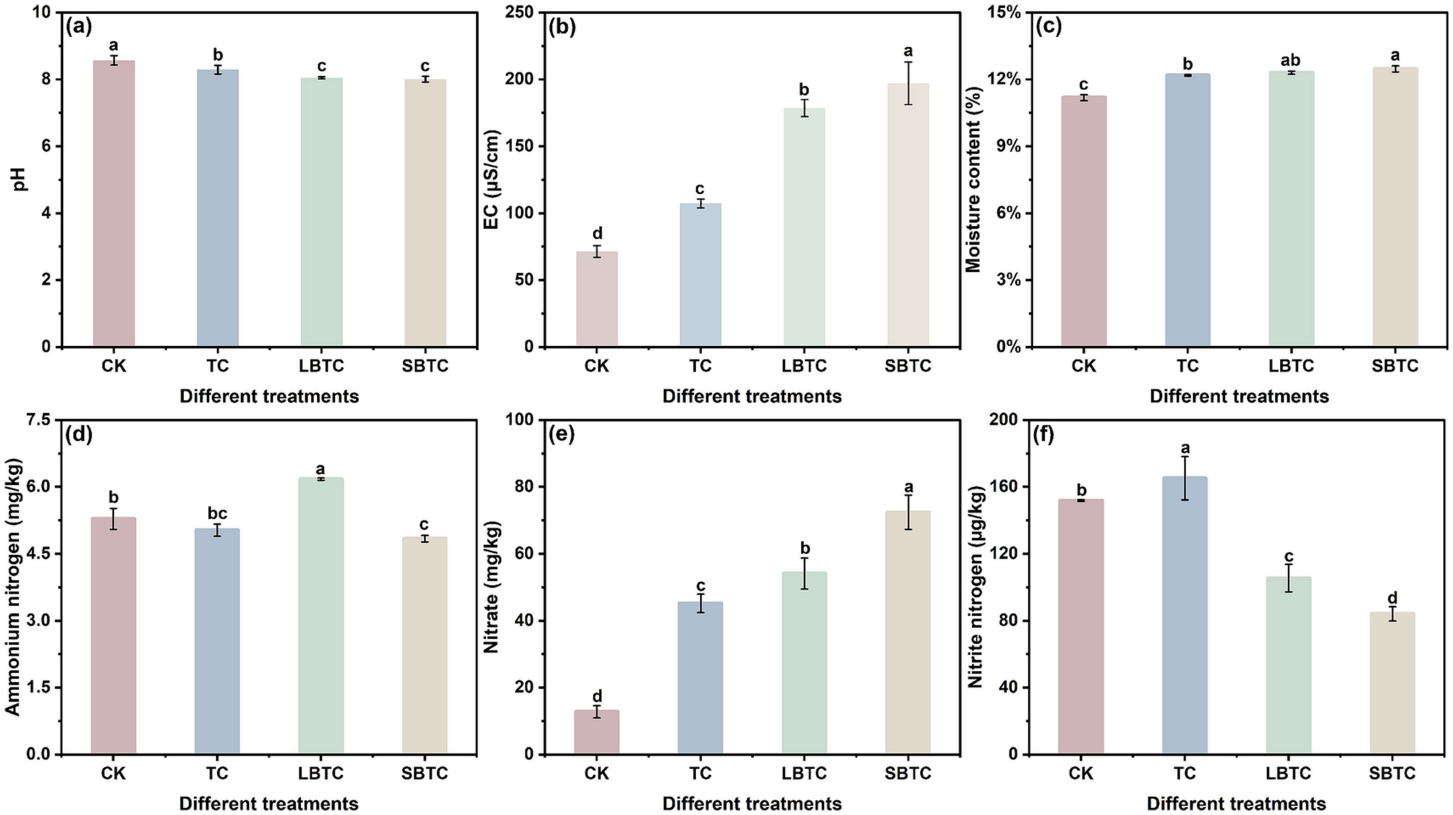
Changes in pH **(A)**, EC **(B)**, moisture content **(C)**, ammonium nitrogen **(D)**, nitrate **(E)**, and nitrite **(F)** in fertilized or unfertilized compost soils. Different letters represent significant differences between groups for the same indicator; *p* < 0.05.

In soils, the application of composts resulted in changes in pH, EC, moisture content, ammonium, nitrate, and nitrite nitrogen (Figure 1). The soil application of TC, LBTC, and SBTC reduced pH by 0.28, 0.52, and 0.56, respectively, compared with that of the CK group (Figure 1A). The application of composts resulted in the entry of exogenous nutrients into soils, which aided in the release of free organic acids, thereby reducing soil pH (Siedt et al., 2021). This condition provided a suitable environment for nutrient uptake by microorganisms (Jiang et al., 2024). The SBTC group, followed by the LBTC, TC, and CK groups, had the highest soil EC (Figure 1B), indicating that compost application improved soil soluble salt content. Compared with that in the CK group, the moisture content in the soils in the TC, LBTC, and SBTC groups had increased by 1, 1.13, and 1.29%, respectively (Figure 1C). The input of exogenous nutrients effectively improved the water retention performance of soils (Chaganti et al., 2015).

In soils, the application of composts decreased ammonium nitrogen content and elevated nitrate content (Figures 1D,E), indicating an enhancement in the vital activities of nitrifying bacteria. Notably, the soil application of composts amended with biochar of different sizes significantly decreased the levels of nitrite (Figure 1F). The presence of biochar with varying sizes in soils promoted the shift from ammonium to nitrate but inhibited the production of nitrite nitrogen. Biochar with high porosity and a large specific surface area could promote nitrification by enhancing soil aeration and increasing the adsorption of nitrification inhibitors (Liu et al., 2024).

### 3.2 Changes in soil nutrient levels

The application of composts to the soil significantly enhanced the content of organic matter, total nitrogen, total phosphorus, total potassium, available nitrogen, available phosphorus, and available potassium compared to the CK group (*p* < 0.05) (Table 1). The SBTC group, followed by the LBTC, TC, and CK groups, had the highest levels of soil nutrient content. In addition to the input of exogenous organic matter, high water content has a protective mechanism for the decomposition of organic matter in soils (Luo et al., 2016), thus increasing organic matter content. A previous study found that the combination of compost application and high soil moisture content further enhanced the protection of the organic matter (Dijkstra and Keitel, 2024). The high levels of nitrogen, phosphorus, and potassium mean that soils have a high capacity to supply nutrients (Liu et al., 2023). Compared to total nutrients (22–77% increase), available nutrients showed a more substantial increase (36–328%) in soils fertilized with composts (Table 1). The availability and retention of soil nutrients could be significantly improved by compost application (Li et al., 2021). The inclusion of biochar with various sizes during composting improved the properties of the resulting composts, with SBTC demonstrating a more significant effect than LBTC.

**Table 1:** Changes in nutrient contents in fertilized or unfertilized compost soils.

Nutrient indicators	Different fertilization groups			
	CK group	TC group	LBTC group	SBTC group
Organic matter (g/kg)	23.48 ± 0.39^d^	28.12 ± 0.84^c^	31.78 ± 0.94^b^	33.53 ± 0.55^a^
Total nitrogen (g/kg)	1.01 ± 0.02^d^	1.23 ± 0.03^c^	1.46 ± 0.11^b^	1.60 ± 0.06^a^
Total phosphorus (g/kg)	0.90 ± 0.05^c^	1.42 ± 0.09^b^	1.56 ± 0.03^a^	1.60 ± 0.06^a^
Total potassium (g/kg)	15.86 ± 0.07^d^	16.21 ± 0.10^c^	16.60 ± 0.13^b^	17.05 ± 0.21^a^
Available nitrogen (mg/kg)	46.03 ± 4.33^d^	63.00 ± 4.15^c^	82.25 ± 2.45^b^	109.55 ± 15.40^a^
Available phosphorus (mg/kg)	30.15 ± 8.35^d^	66.75 ± 12.75^c^	105.40 ± 9.40^b^	129.20 ± 7.85^a^
Available potassium (mg/kg)	214.2 ± 28.05^d^	310.29 ± 36.06^c^	539.68 ± 50.09^b^	619.82 ± 10.02^a^

Data presented as mean ± standard deviation (*n* = 3); means in the same row followed by different letters indicate significant differences (*p* < 0.05); CK group: soils without composts application; TC group: soils with traditional composts application; LBTC group: soils with large size biochar-amended compost application; SBTC group: soils with small size biochar-amended compost application.

### 3.3 Changes in cucumber qualities

**Figure 2:**
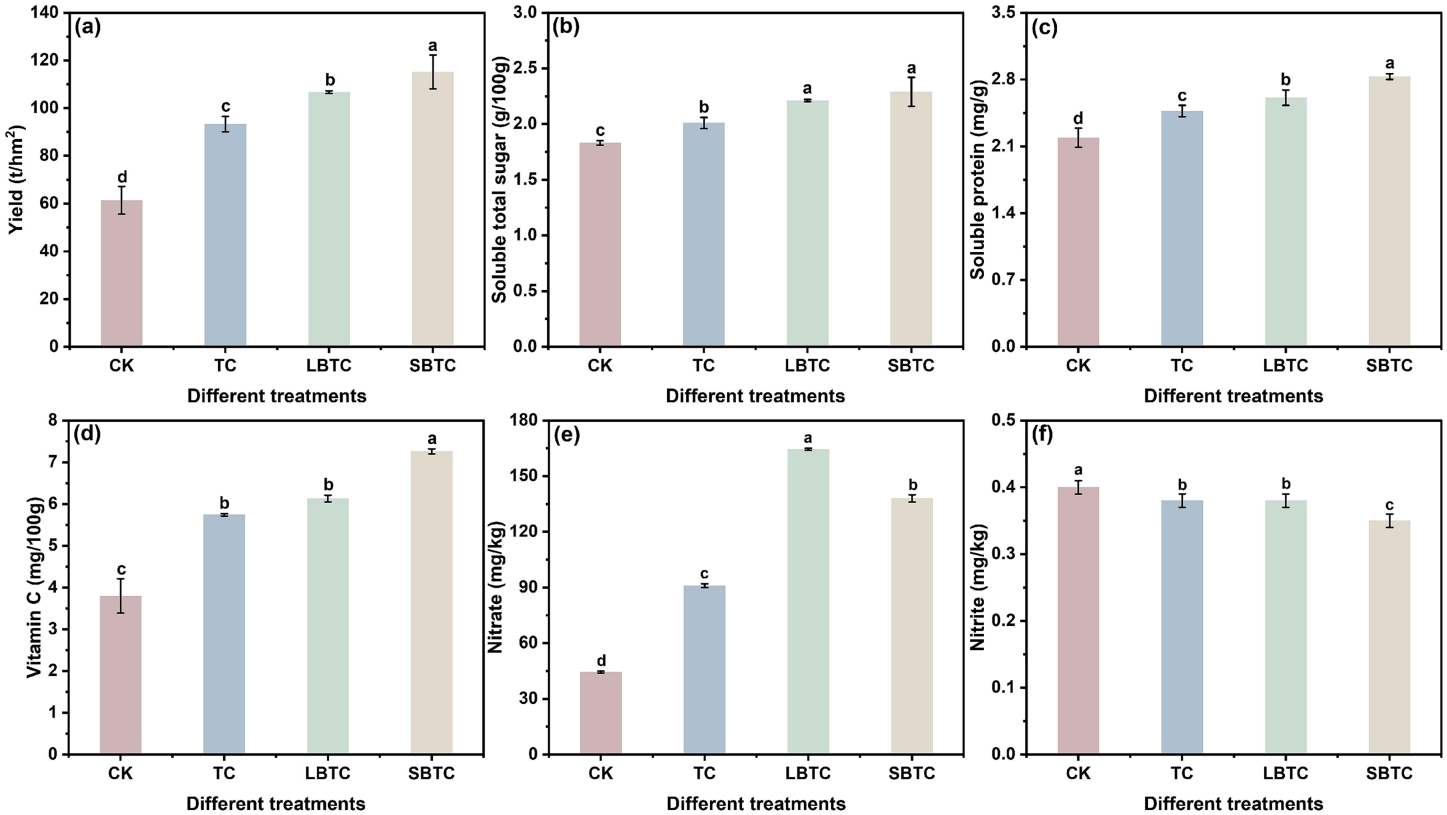
Changes in yield **(A)**, soluble total sugar **(B)**, soluble protein **(C)**, vitamin C **(D)**, nitrate **(E)**, and nitrite **(F)** of cucumber. Different letters represent significant differences between groups for the same indicator; *p* < 0.05.

Figure 2 illustrates that soil compost application significantly improved cucumber yield and quality. The yield, soluble total sugar, soluble protein, and vitamin C levels in cucumbers were highest in the SBTC group, followed by those in the LBTC, TC, and CK groups (Figures 2A–D). Compost application resulted in humus into soils, which remarkably promoted the growth of cucumbers (Jiang et al., 2022). Composts can gradually release nutrients into soils (Siedt et al., 2021). Soils with increased fertility provide a suitable environment for microbial life activities, thereby promoting nutrient cycling and increasing crop yields (Fan et al., 2021). The high specific surface area and porosity of biochar contributed to detoxify the harmful effects of successive cropping on soils (Yang et al., 2019).

Notably, the soil application of composts significantly elevated nitrate levels in cucumbers (*p* < 0.05) (Figure 2E), but significantly decreased nitrite levels (*p* < 0.05) (Figure 2F). The accumulation of nitrate in soils can enhance the spread of nitrate to plants (Yang et al., 2024). Nitrate nitrogen was significantly higher in soils in the SBTC group than in those in the LBTC group but was low in cucumbers. A previous study found that biochar significantly increased the retention of nitrate in soils (Cooper et al., 2023). SBTC, which contains biochar with a small size and high specific surface area, might be favorable for the retention of nitrate in soils, thereby inhibiting its spread to plants. The low levels of nitrite in soils showed an important contribution to the low accumulation of nitrite in cucumbers (Figure 1F). In addition, elevated nitrogen nutrients in soils were an important factor in the reduction in nitrite content in cucumbers (Zhang W. et al., 2023; Zhang X. et al., 2023). Adequate nitrogen content in soils can promote photosynthesis in cucumber leaves, which in turn promotes the accumulation of nitrite reductase in cucumber leaves (Szal and Podgórska, 2012). This effect increased the reduction of nitrite in cucumber leaves into ammonia nitrogen, thereby reducing nitrite transmission from leaves to cucumbers. Therefore, the application of SBTC to soils, followed by that of LBTC and TC, was the most effective way for reducing nitrite content in cucumbers.

### 3.4 Changes in the structure of soil bacterial communities

**Figure 3:**
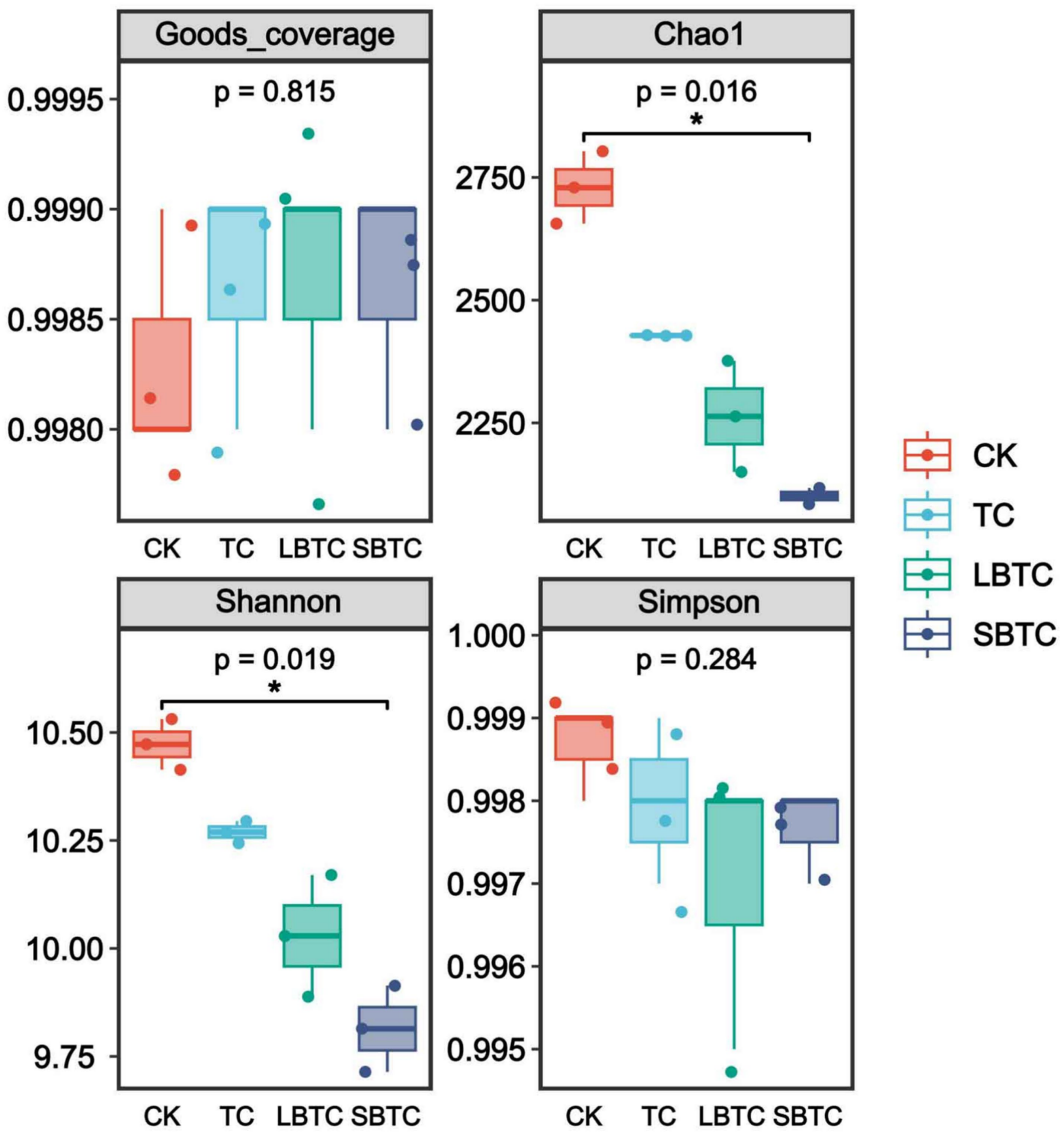
Changes in microbial diversity indexes in fertilized or unfertilized compost soils. *p*-values indicate overall differences between groups based on Kruskal–Wallis non-parametric test; markers for significance levels indicate differences obtained from two-by-two comparisons between groups based on Dunn’s test; *p < 0.05; **p < 0.01; ***p < 0.001.

Figure 3 illustrates that in the soil samples of all groups, the Good’s coverage index was above 0.99, indicating that the distribution of bacterial communities was accurately represented. The Simpson index did not show significant differences in the soils of different groups. Notably, compared with those of the CK group, the Chao1 and Shannon indices of soils fertilized with composts had significantly decreased (*p* < 0.05), indicating that compost application decreased soil microbial richness and diversity (Wang et al., 2024). However, the above results were inconsistent with the findings of a previous study, which reported that compost application significantly improved the *α*-diversity of soil bacterial communities (Wu et al., 2020). This effect might be primarily related to the reduction in soil pH caused by soil compost application (Figure 1A). Another study also found that the α-diversity of bacterial communities had a significant positive correlation with soil pH (Zhang W. et al., 2023; Zhang X. et al., 2023). A low bacterial diversity indicates that the density of bacteria in soils is relatively low and the potential for mutual contact between bacteria has decreased, creating favorable conditions for reducing the horizontal gene transfer of ARGs (Zhu et al., 2023), and thus facilitating the removal of ARGs from soil.

**Figure 4:**
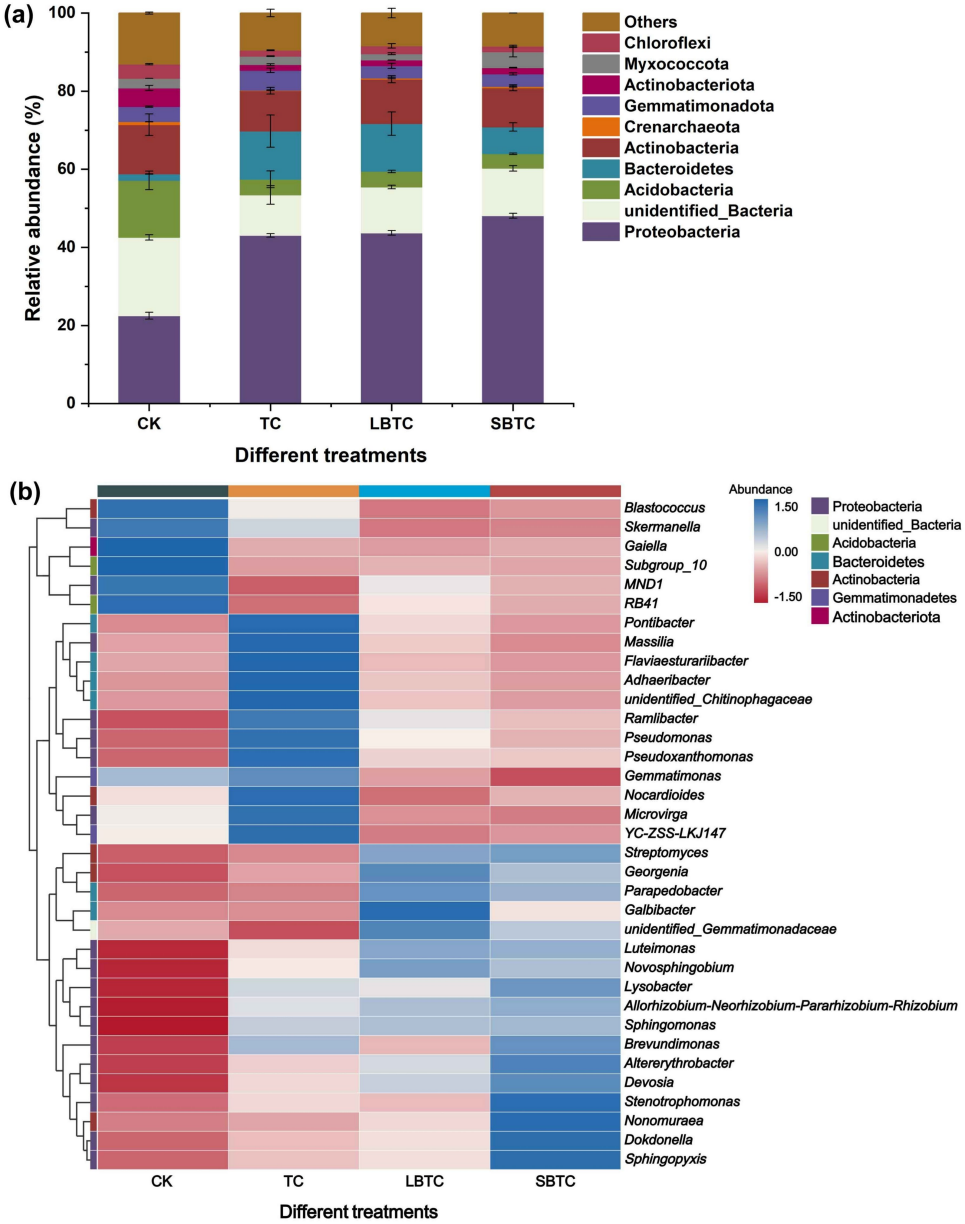
Changes in bacterial communities in fertilized or unfertilized compost soils. Relative abundance of the phylum-level bacteria **(A)** and the top 35 genus-level bacteria **(B)**. The abundance bar indicates the result of *Z*-score normalization of the data, and larger values indicate a higher abundance of the corresponding genus-level microorganisms; the blue color represents high abundance; and the red color represents low abundance.

In soils, the dominant bacterial phyla were Proteobacteria, Acidobacteria, Actinobacteria, and Bacteroidetes, which accounted for 22.49–48.08%, 3.72–14.61%, 1.65–12.33%, and 10.01–12.59% of the total bacterial communities, respectively (Figure 4A). The dominant bacterial phyla were Firmicutes, Actinobacteria, Proteobacteria, Bacteroidetes, and Acidobacteria in the soils before this experiment (Tong et al., 2023a). They demonstrate that the high-dose (18.73 t/ha) application of compost to the soil altered the community structure of native soil bacteria. Only the relative abundances of Proteobacteria and Bacteroidetes significantly increased (*p* < 0.05), while those of all other bacterial phyla significantly decreased (*p* < 0.05) in soils fertilized with compost compared to those in the CK group. This reduction was the primary reason for the decline in soil microbial richness and diversity (Figure 3). Proteobacteria and Bacteroidetes play a crucial driving role in nutrient cycling in soils (Luo et al., 2022). The enhancement of their metabolic activities increased the availability of nutrients in the soil (Kuppusamy et al., 2023), boosting soil fertility while promoting plant growth. Previous studies have shown that compost application reduces soil bulk density and enhances soil porosity (Seyedsadr et al., 2022) and that Proteobacteria is suitable for growth and reproduction in well-aerated conditions (Tashiro et al., 2018). The abundances of Bacteroidetes (7–12%) and Proteobacteria (43–48%) in the compost-treated soils in this study were higher than those in a previous report (approximately 8%) (Tang et al., 2023). This difference might be due to the high nutrient content of the composts, especially the biochar-amended composts, used in the present study. Proteobacteria and Bacteroidetes, as eutrophic bacteria, tend to inhabit soils with high nutrient contents (Zhang et al., 2021). In contrast, Acidobacteria is an oligotrophic bacterium (Wang et al., 2020) and is thus unsuitable for growth in soils fertilized with compost. In soils, compost application resulted in the direct or slow release of a large amount of nutrients, thereby changing the bacterial community structure. Actinobacteria is a major producer of antibiotics and a key host of ARGs (Feng et al., 2023). The decrease in its relative abundance by compost application might have a positive effect on ARG removal from soils.

Figure 4B shows that among the top 35 bacterial genera in soils in terms of relative abundance, 18 and six bacterial genera belonged to Proteobacteria and Bacteroidetes phyla, respectively. This finding revealed that the distribution of dominant bacterial genera in soils before this experiment was considerably different, with 12 and 13 bacterial genera belonging to the Firmicutes and Proteobacteria phyla, respectively (Tong et al., 2023a,b). In soils, the high-dose (18.73 t/ha) application of composts substantially inhibited the life activities of bacterial genera in Firmicutes phylum. This finding is also a positive indication for the removal of ARGs from soils, as bacterial genera from the Firmicutes phylum were the dominant potential host bacteria for ARGs in TC, LBTC, and SBTC, accounting for above 50% of the total number of potential host bacteria (Tong et al., 2023b). The relative abundance of *Sphingomonas*, the predominant bacterial genus belonging to the Proteobacteria phylum, increased by 45, 49, and 51% in TC, LBTC, and SBTC groups than that of the CK group (Figure 4B). *Sphingomonas* shows a strong ability to promote plant growth and inhibit the occurrence of plant diseases (Hernández-Lara et al., 2023). Biochar further enhanced the dominance of bacteria belonging to Proteobacteria in soils (Li H. et al., 2022; Li X. et al., 2022). In addition to supplying nutrients, the small-sized biochar in SBTC had a larger specific surface area and more pores than the large-sized biochar in LBTC, creating a suitable environment for bacteria to survive and promoting plant growth as a result. *Lysobacter*, *Pontibacter*, *Devosia*, *Luteimonas*, *Dokdonella*, *Sphingopyxis*, and *Novosphingobium*, which are members of Proteobacteria, also showed a high abundance in soils fertilized with composts amended with biochar with different sizes (Figure 4B). The relative abundance of *Streptomyces*, a member of the Actinobacteria phylum, increased by 20, 127, and 135% in the TC, LBTC, and SBTC groups, respectively, relative to the CK group. It is also a beneficial bacteria that promotes plant growth (Wu et al., 2021). Meanwhile, a previous study found that composts with higher maturity showed a more dramatic effect on bacterial communities in soils than those with lower maturity (Luo et al., 2022). Compost maturity followed the order: SBTC > LBTC > TC (Tong et al., 2023b). This order was consistent with the effect of the composts on the bacterial communities in soils. The high-dose (18.73 t/ha) application of composts to the soil enhanced cucumber growth and improved soil nutrient availability by promoting the essential activities of beneficial bacteria.

### 3.5 Changes in the absolute abundances of antibiotic resistance genes and mobile genetic elements in soils

**Figure 5:**
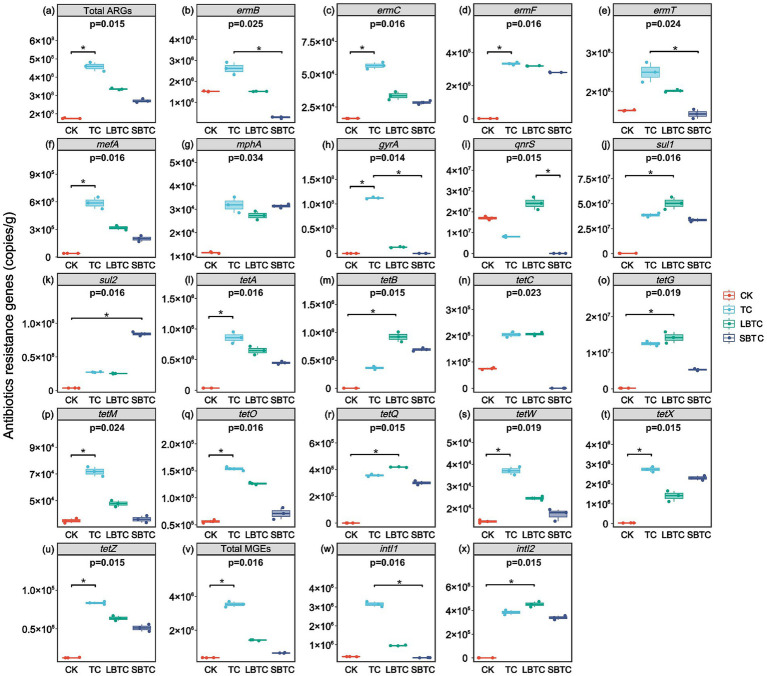
Changes in antibiotics resistance genes and mobile genetic elements in fertilized or unfertilized compost soils. Total ARGs **(A)**, *ermB* **(B)**, *ermC* **(C)**, *ermF* **(D)**, *ermT* **(E)**, *mefA* **(F)**, *mphA* **(G)**, *gyrA* **(H)**, *qnrS* **(I)**, *sul1* **(J)**, *sul2* **(K)**, *tetA* **(L)**, *tetB* **(M)**, *tetC* **(N)**, *tetG* **(O)**, *tetM* **(P)**, *tetO* **(Q)**, *tetQ* **(R)**, *tetW* **(S)**, *tetX* **(T)**, *tetZ* **(U)**, total MGEs **(V)**, *intI1* **(W)**, and *intI2* **(X)**. *p*-values indicate overall differences between groups based on Kruskal–Wallis non-parametric test; markers for significance levels indicate differences obtained from two-by-two comparisons between groups based on Dunn’s test; *p < 0.05; **p < 0.01; ***p < 0.001.

Figure 5 shows that the high-dose (18.73 t/ha) application of composts to the soil significantly changed the absolute abundances of ARGs in soils. Compared with that in the CK group (1.75 × 10^8^ copies/g), the total absolute abundances of ARGs in soils had increased by 161, 91, and 55% in the TC (4.57 × 10^8^ copies/g), LBTC (3.35 × 10^8^ copies/g), and SBTC (2.71 × 10^8^ copies/g) groups, respectively (Figure 5A). However, a previous study found that the application of manure composts to soils with a 26-year history of fertilization resulted in a decrease in the abundance of ARGs (Xu et al., 2019). The soils in this study had a brief history of compost application of only 2 years, which resulted in the limited effectiveness of compost application in removing ARGs from soils. However, the total absolute abundances of ARGs in soils increased by 23.88, 5.59, 5.50, and 5.49 times in the CK, TC, LBTC, and SBTC groups, respectively, compared to their preexperimental values. Cucumber cultivation promoted the proliferation of ARGs in soils. The high-dose (18.73 t/ha) application of composts to the soil is an effective strategy that inhibited the proliferation of ARGs, with SBTC showing the most significant effect. Although the soil application of composts increased ARGs in the short term, it gradually decreased ARGs over time (Zhang et al., 2018). The high-dose (18.73 t/ha) application of SBTC to the soil also resulted in part of the ARGs (such as *ermB*, *ermT*, *gyrA*, *qnrS*, *tetC*, and *tetM*) showing a similar trend. Compared with those in the CK group, the absolute abundances of *ermB*, *ermT*, *qnrS*, and *tetC* in soils decreased by 82, 6, 100, and 100% in the SBTC group, respectively (Figures 5B,E,I,N). In the CK and SBTC groups, the gene *gyrA* was not detected in soils, and the gene *tetM* in soils showed no significant difference in terms of absolute abundance (*p* > 0.05) (Figures 5H,P). The absolute abundance of *ermB* in soils also showed no significant difference (*p* > 0.05) in the CK and LBTC groups (Figure 5B). The input of antibiotics from composts into soils was able to continuously induce the proliferation of ARGs (Cerqueira et al., 2020). The composts amended with biochar with different sizes contained lower levels of antibiotics than TC (Tong et al., 2023b), thus decreasing the amplification pressure on ARGs in soils. The increase in the diversity of bacterial communities led to an increase in the diversities of native ARGs in soils (Wang et al., 2018). The decrease in bacterial community diversities in soils applied with composts demonstrates the positive effect on ARGs removal in soils. Exogenously inputted ARGs do not persist in soils and the risk of their spread in soils is low (Barrios et al., 2020). Changes in soil pH and nutrient availability can inhibit the growth of antibiotic-resistant bacteria (Chen et al., 2017), thereby decreasing the abundance of ARGs in soils.

The application of composts significantly changed the absolute abundance of MGEs, which are important mediators of horizontal gene transfer by ARGs. Compared with those in the CK group (3.70 × 10^5^ copies/g), the total absolute abundances of MGEs in soils increased by 8.55, 2.81, and 0.76 times in the TC (3.53 × 10^6^ copies/g), LBTC (1.41 × 10^6^ copies/g), and SBTC (6.49 × 10^5^ copies/g) groups, respectively (Figure 5V). The changes in MGEs in soils were consistent with the changes in ARGs in soils, which also showed a short-lived increase in soils fertilized with compost. The total absolute abundances of MGEs increased by 6.66, 5.23, 1.81, and 0.47 times in the CK, TC, LBTC, and SBTC groups, respectively, relative to their preexperimental values. The high-dose (18.73 t/ha) application of composts to the soil can better control the proliferation of MGEs in soils than that of ARGs. In this study, MGEs mainly consisted of *intI1* and *intI2* (Figures 5W,X). Integrase genes effectively represent the horizontal gene transfer frequency of ARGs (Zhu et al., 2013). The planting of cucumbers promoted the horizontal gene transfer of ARGs in soils; this process was inhibited by the high-dose (18.73 t/ha) application of compost to the soil. Meanwhile, the high-dose (18.73 t/ha) application of SBTC to the soil decreased the absolute abundance of *intI1* by 15.67%, indicating that the probability of transferring ARGs from compost-derived bacteria to native soil bacteria had reduced. Biochar with porous structures increases the distance between microbes and decreases the possibility of mutual contact between them (Shao et al., 2022). Mutual contact between microorganisms is an important factor in the horizontal gene transfer of ARGs (Martínez et al., 2015). The small-sized biochar with numerous pore structures can inhibit the horizontal gene transfer of ARGs well (Tong et al., 2023b). Meanwhile, biochar showed increased pores and reduced pore size after being composted (Hagemann et al., 2017), enhancing the inhibitory effects described above. The *intI1* and *intI2* exhibited a significant positive correlation with most ARGs (Supplementary Figure S1). This correlation indicated that the high-dose (18.73 t/ha) application of composts inhibited the increase in the abundance of MGEs in soils, which in turn inhibited the horizontal gene transfer of ARGs, thereby decreasing the proliferation pressure of ARGs in soils.

### 3.6 Distribution of the potential host bacteria of antibiotic resistance genes in soils

**Figure 6:**
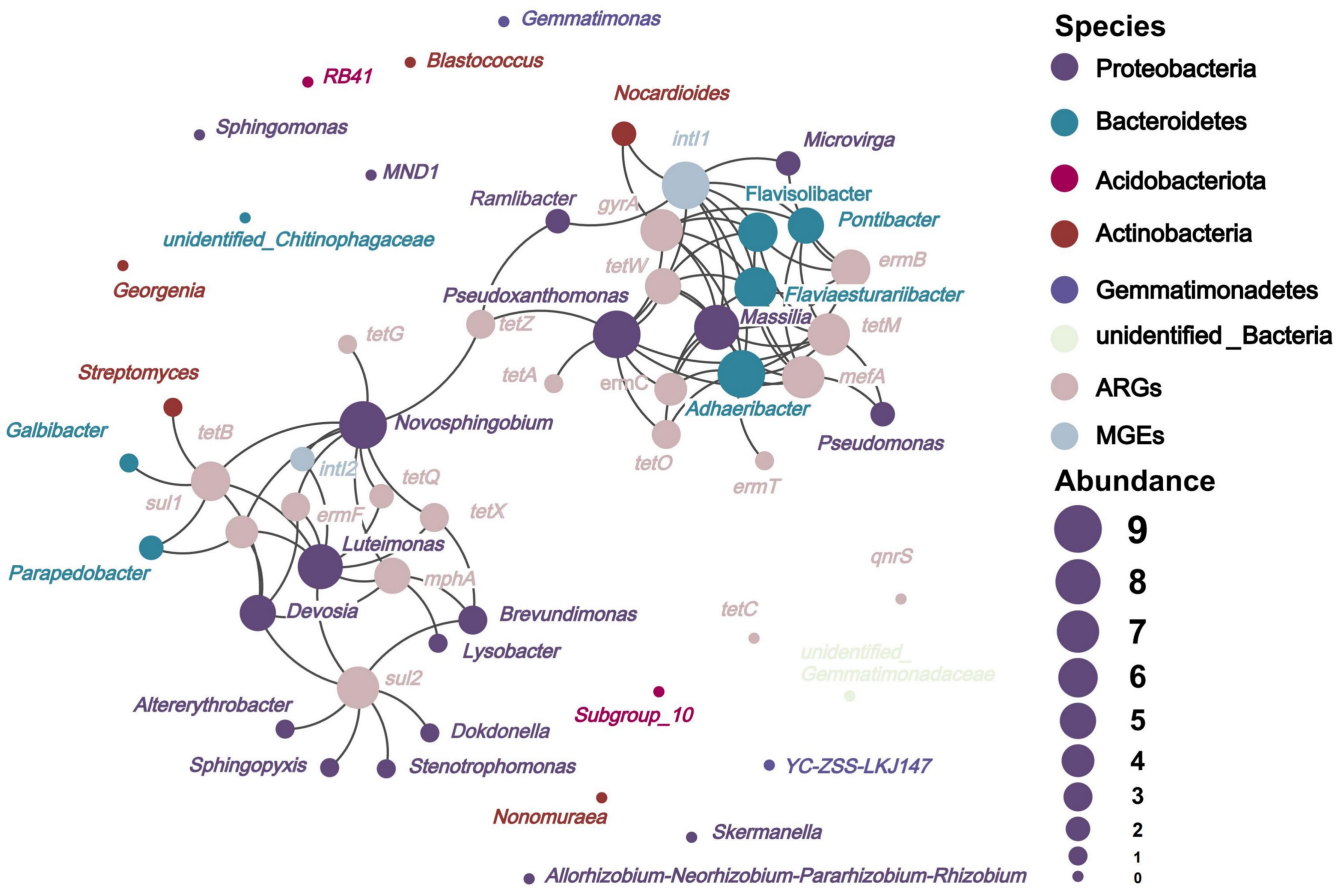
Network analysis revealed potential host bacterial distribution of ARGs and MGEs in soils. The circle size indicates the abundance of the number of nodes connected to other nodes; the larger the circle size indicates that the node is connected to more other nodes; the line between different nodes indicates a significant positive correlation; *p* < 0.05.

Figure 6 depicts that on the basis of network analysis, 22 species of potential bacterial hosts of ARGs and MGEs were identified at the genus level in soils applied with composts. They mainly belonged to Proteobacteria, Bacteroidetes, and Actinobacteria, which were different from the main host bacterial phyla, namely, Firmicutes, Actinobacteria, Proteobacteria, and Bacteroidetes, in the soils before the experiment (Tong et al., 2023a). The high-dose (18.73 t/ha) application of composts to the soil inhibited the spread of ARGs between different bacterial phyla in soils. Figure 6 shows that *Adhaeribacter* (carrying *ermB*, *ermC*, *ermT*, *mefA*, *gyrA*, *tetM*, *tetO*, and *tetW*), *Devosia* (carrying *ermF*, *mphA*, *sul1*, *sul2*, and *tetB*), *Flaviaesturariibacter* (carrying *ermB*, *ermC*, *mefA*, *gyrA*, *tetM*, and *tetW*), *Flavisolibacter* (carrying *ermB*, *mefA*, *gyrA*, *tetM*, and *tetW*), *Luteimonas* (carrying *ermF*, *mphA*, *sul1*, *sul2*, *tetB*, *tetQ*, and *tetX*), *Massilia* (carrying *ermB*, *ermC*, *mefA*, *gyrA*, *tetM*, *tetO*, and *tetW*), *Novosphingobium* (carrying *ermF*, *mphA*, *sul1*, *tetB*, *tetG*, *tetQ*, *tetX*, and *tetZ*), and *Pseudoxanthomonas* (carrying *ermC*, *mefA*, *gyrA*, *tetA*, *tetM*, *tetO*, *tetW*, *tetZ*) harbored five to eight types of ARGs. These bacterial genera are the important potential hosts of ARGs in soils. They might be more effective in facilitating the spread of ARGs in the environment than other potential host bacteria (Buta-Hubeny et al., 2022). The *ermT*, *tetA*, *tetG*, and *tetQ* are hosted by only one or two bacterial species. However, other ARGs were hosted by more than two types of bacteria. The high diversity of hosts enhances the persistence of their associated ARGs in soils (Zhang et al., 2018), making the removal of ARGs difficult.

The potential host bacteria of *intI1* and *intI2* were *Pontibacter*, *Massilia*, *Adhaeribacter*, *Flaviaesturariibacter*, *Ramlibacter*, *Pseudoxanthomonas*, *Microvirga*, *Nocardioides*, *Flavisolibacter*, *Luteimonas*, and *Novosphingobium*, which were the hubs for the horizontal gene transfer of ARGs. ARGs can persist in soils with the assistance of horizontal gene transfer (Li and Zhang, 2022). Normally, the abundance of ARGs varies with the abundance of bacteria (Wang et al., 2023), especially those of host bacteria. The abundances of *Massilia*, *Adhaeribacter*, *Flaviaesturariibacter*, *Microvirga*, and *Nocardioides*, the potential hosts of ARGs and MGEs, decreased in soils fertilized with composts amended with biochar with different sizes (Figure 4B). This effect contributed to the inhibition of the horizontal gene transfer of ARGs and further facilitated their removal from soils.

### 3.7 Changes in the relative abundance of antibiotic resistance genes in cucumbers

**Figure 7:**
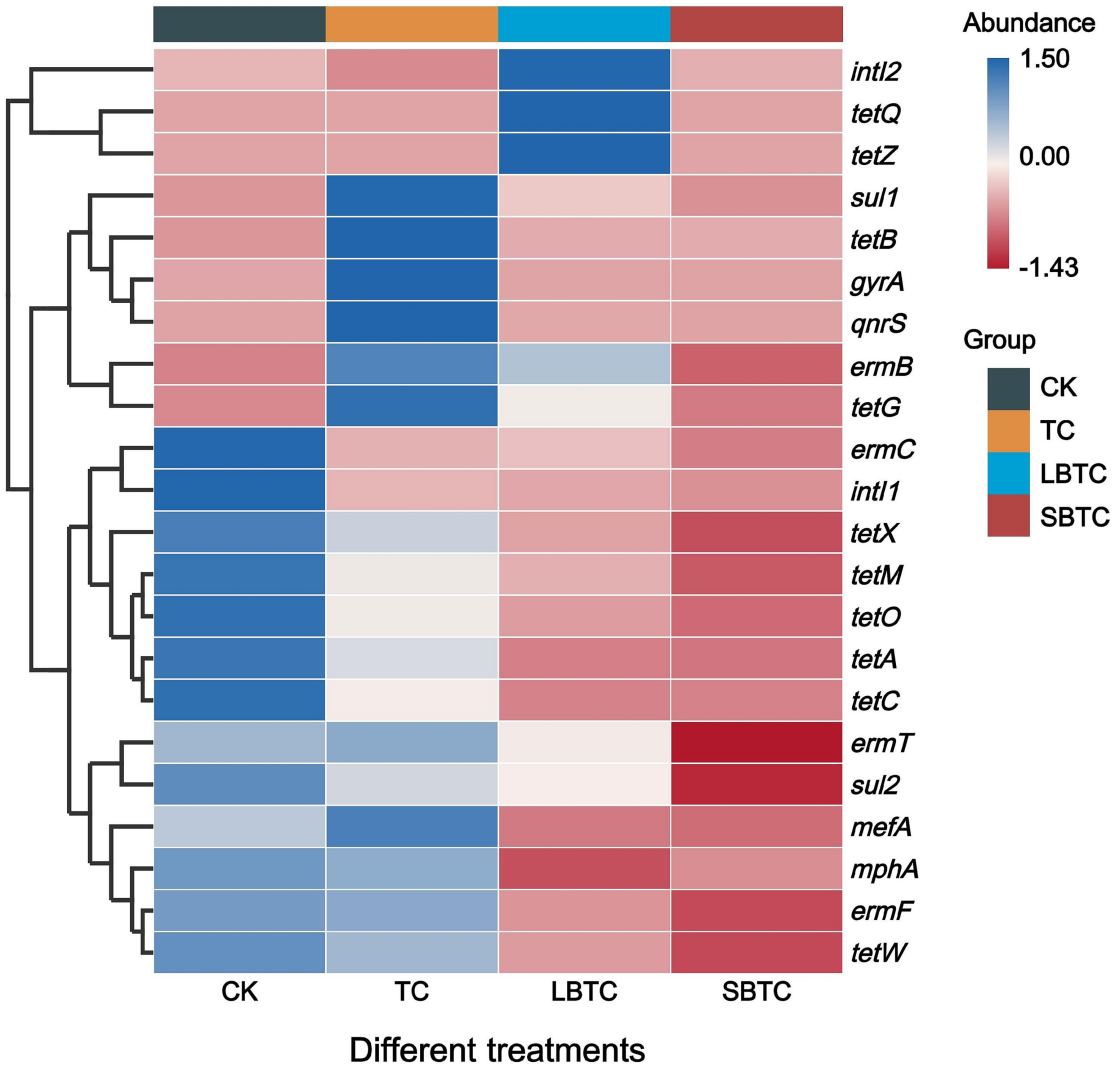
Changes in the relative abundance of ARGs and MGEs in cucumbers. The abundance bar indicates the result of *Z*-score normalization of the data, and larger values indicate a higher abundance of the corresponding ARGs; the blue color represents high abundance; and the red color represents low abundance.

Figure 7 shows that the relative abundance of ARGs and MGEs in cucumbers changed through the application of composts to soils. Compared with those in the CK group, the relative abundance of *ermC*, *ermF*, *mphA*, *sul2*, *tetA*, *tetC*, *tetM*, *tetO*, *tetW*, and *tetX* had decreased by 4.40–64.54%, 23.32–100%, and 57.29–100% in the cucumbers in the TC, LBTC, and SBTC groups, respectively. SBTC decreased the relative abundances of *ermB*, *ermT*, *mefA*, *gyrA*, *sul1*, and *tetG* in cucumbers by 68.29, 84.28, 66.13, 86.12, 14.67, and 9.55%, respectively. Meanwhile, LBTC decreased the relative abundances of *ermT*, *mefA*, and *gyrA* in cucumbers by 23.32, 61.67, and 56.09%, respectively. The *tetQ* and *tetZ* were not detected in the cucumbers in the CK, TC, and SBTC groups. This result was inconsistent with the previous finding showing that the soil application of composts amended with biochar with different sizes only decreased *sul1* and *tetG* abundance in cucumbers (Tong et al., 2023a). Another study found that the high accumulation of ARGs in soils due to the soil application of organic fertilizer promoted the spread of ARGs from soils into plants (Sun et al., 2021). The high-dose (18.73 t/ha) application of composts to the soil inhibited the proliferation of ARGs by reducing the life activities of host bacteria in soils, thereby reducing the spread of ARGs from soils to cucumbers. The microorganisms carrying ARGs in soils spread through roots into other tissues of plants (Chen et al., 2018). They can colonize the surface of biochar due to its porous structure and nutritional benefits (Qi et al., 2022). Biochar might also absorb the host bacteria of ARGs, thereby suppressing the spread of ARGs from soils to cucumbers. Compared with that of LBTC, the high-dose (18.73 t/ha) application of SBTC to the soil showed a better effect on the removal of ARGs in cucumbers. A previous study found that ARGs can be effectively removed from fertilized biochar soils (Fu et al., 2021). Moreover, biochar with small sizes improved the removal of ARGs from soils (Su et al., 2024). The high-dose (18.73 t/ha) application of SBTC to the soil provided favorable soil conditions for decreasing the spread of ARGs from soils to cucumbers.

Compared with that in the cucumbers in the CK group, the relative abundances of *intI1* decreased by 86.78, 91.42, and 98.03% in the cucumbers in the TC, LBTC, and SBTC groups, respectively, and the relative abundances of *intI2* decreased by 100 and 11.97% in the cucumbers in the TC and SBTC groups, respectively (Figure 7). The high-dose (18.73 t/ha) soil application of composts to the soil inhibited the horizontal gene transfer of ARGs in cucumbers, thus reducing ARGs proliferation. This finding was inconsistent with the result of a previous study reporting that compost application promoted *intI1* accumulation in soils while promoting *intI1* accumulation in plants (Xu et al., 2021) likely because the application of biochar-amended and non-biochar-amended composts in this study increased the electrical conductivity of soils (Figure 1B). The increase in salt content in soils can inhibit the horizontal gene transfer of ARGs due to MGEs in soils (Ji and Pan, 2022). This effect provided a good basis for inhibiting the spread of *intI1* from soils to plants. The *intI1* exerted an important effect on the proliferation of ARGs in soil–vegetable systems (Wei et al., 2020). In this study, *intI1* showed significant positive correlations with *ermC*, *ermF*, *mphA*, *sul2*, *tetA*, *tetC*, *tetM*, *tetO*, *tetW*, and *tetX* (*p* < 0.05). In contrast, *intI2* showed a significant positive correlation only with *tetQ* and *tetZ* (*p* < 0.05) (Supplementary Figure S2). The high-dose (18.73 t/ha) application of compost to the soil inhibited the proliferation of MGEs through the inhibition of the life activities of their host bacteria in soils, thus decreasing the transfer of MGEs from soils to cucumbers.

### 3.8 Factors driving the changes in antibiotics resistance genes in the soil–cucumber systems

**Figure 8:**
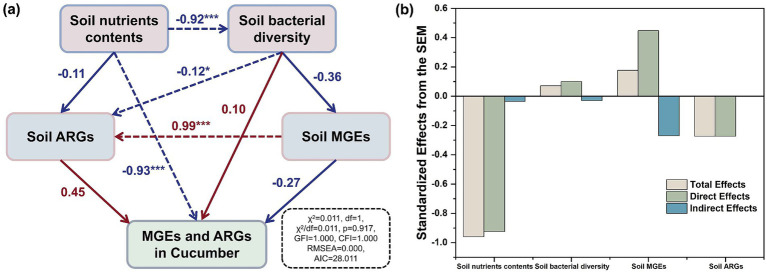
Structural equation model (SEM) evaluated the direct and indirect effects of soil nutrient contents, bacterial diversity, MGEs, and ARGs on the accumulation of MGEs and ARGs in cucumbers **(A)**, and the standardized effects derived from SEM **(B)**. The numbers near the arrows are normalized path coefficients, which are used to indicate the strength of the relationship between the variables; assessment of the fit of the model: (1) a chi-square value to degrees of freedom ratio (*χ*^2^/df), used to test the fit between the model and the data, less than 1; (2) the *p*-value for the chi-square test was greater than 0.05; (3) Goodness-of-fit index (GFI), indicated the overall quality of the model fit, higher than 0.9 and close to 1; (4) Comparison-of-fit index (CFI) higher than 0.9 and close to 1; (5) A mean squared and variance (RMSEA) residual error of less than 0.05; (6) Akaike information criterion (AIC), which is used to compare the strengths and weaknesses of different models, the smaller its value the better the model fit the red and blue arrows represent positive and negative relationships; the dotted lines represent the significant correlations; *p < 0.05, **p < 0.01, ***p < 0.001.

The SEM revealed that under the high-dose (18.73 t/ha) application of compost to the soil, nutrient contents were the key driver of the changes in the ARGs and MGEs in the soil–cucumber system (Figure 8A). As shown in Figure 8B, each factor produced the standardized effects in total, directly, and indirectly from the SEM. Soil nutrient contents had a significant negative effect on soil bacterial diversity (*λ* = −0.92, *p* < 0.001) mainly because their increase resulted in the growth of Proteobacteria and Bacteroidetes and the inhibition of other bacterial phyla (Figure 4A). A previous study also found that the application of highly mature composts helped in the removal of pathogenic bacteria and fungal growth from soils (Luo et al., 2022). Microbial communities are critical for the variation in ARGs (Forsberg et al., 2014). Meanwhile, soil bacterial diversity showed a significant effect on ARGs in soils (*λ* = −0.12, *p <* 0.05). The fate of ARGs mainly depended on their host bacteria (Zheng et al., 2020). In soils, MGEs showed a significant positive effect on ARGs (*λ* = 0.99, *p* < 0.001). This finding was consistent with the results of previous studies showing that in soils, MGEs contributed more than bacterial communities to changes in ARGs (Li H. et al., 2022; Li X. et al., 2022; Hu et al., 2017). In soils, the variation in MGEs was similar to that of ARGs, further supporting the above view. The decrease in the horizontal gene transfer of ARGs in soils through the inhibition of the proliferation of MGEs was effective in controlling the proliferation of ARGs (Zheng et al., 2021).

Moreover, the accumulation of MGEs and ARGs in soils due to the high-dose (18.73 t/ha) application of composts to the soil did not promote the transfer of MGEs and ARGs into cucumbers. They showed no significant effect on the MGEs and ARGs in cucumbers (*λ* = −0.27 and 0.45, *p* > 0.05). The increase in soil nutrient contents resulted in only a transient increase in ARGs, as the high dose (18.73 t/ha) of composts applied to soils inhibited the proliferation of ARGs compared to the soils before this experiment. The increase in the nutrient contents in soils fertilized with composts mainly promoted the growth of bacteria that are beneficial to plant growth and soil nutrient cycling (Jiang et al., 2024; Bello et al., 2023). It resulted in the decreased competitiveness of the host bacteria of ARGs and MGEs in soils, thus inhibiting their transfer into cucumbers. Redundancy analysis showed that among the nutrient indicators, total phosphorus had the highest contribution of 68.6% to the variation in ARGs and MGEs in cucumbers, followed by available potassium, which had a contribution of 24.5% (Supplementary Figure S3A). Meanwhile, redundancy analysis also showed that among the nutrient indicators, total phosphorus had the highest contribution of 93.3% to the variation in cucumber yield and quality indicators (Supplementary Figure S3B). This finding suggests that total phosphorus is the key nutrient indicator for inhibiting the spread of ARGs and MGEs from soils to cucumbers by promoting cucumber growth. A previous study found that the increased content and availability of phosphorus nutrients can inhibit the environmental spread of pathogenic bacteria in soils, especially in plants (Cao et al., 2024). At the same time, the increased available potassium content of soils can inhibit the occurrence of cucumber diseases, that is, the spread of pathogenic bacteria from soils into cucumbers was inhibited (Li et al., 2023). Pathogenic bacteria are well known to be key hosts of ARGs (Yu et al., 2023). Therefore, enhancing the content and availability of phosphorus and potassium created a healthy soil base for preventing the spread of ARGs from soils to cucumbers. In addition, a previous study found that increased nutrient content in soils can decrease the frequency of ARG transfer from soils to plant endophytes (Xu et al., 2024). The increase in soil nutrient contents decreased the abundance of MGEs in cucumbers, therefore reducing the accumulation of ARGs in cucumbers. The above results indicate that the high-dose (18.73 t/ha) application of composts to the soil did not promote the transfer of MGEs and ARGs from soils to cucumbers by increasing soil nutrient contents, but instead inhibited the transfer of ARGs and MGEs from soils to cucumbers by suppressing the proliferation of ARGs and MGEs in soils.

## 4 Conclusion

The high-dose (18.73 t/ha) application of compost in soil improved levels of soil nutrient contents, and cucumber yield and quality, with SBTC showing the best effect. The high-dose (18.73 t/ha) application of composts to the soil also caused the accumulation of ARGs in soils. However, it inhibited the proliferation of ARGs and MGEs in soils compared to the soils before this experiment, further decreasing their abundance in cucumber. The application of SBTC significantly impacted the bacterial communities and reduced the abundance of *intI1* in soils. The accumulation and distribution of ARGs in the soil–cucumber system mainly depended on nutrients, MGEs, and their host bacteria in the soil. The increase in soil nutrient contents enhanced the competitiveness of bacteria that promoted cucumber growth and soil nutrient availability, thereby inhibiting the vital activities of the host bacteria of ARGs and MGEs. In summary, the high-dose (18.73 t/ha) application of SBTC to the soil is more effective than the other groups in reducing the risk of ARGs in the investigated soil–cucumber system.

## Data Availability

The raw data supporting the conclusions of this article will be made available by the authors, without undue reservation.
