# Supplementary material for: Application of compost amended with biochar on the distribution of antibiotic resistance genes in a soil–cucumber system—from the perspective of high-dose fertilization
Source: Front Microbiol. 2025 Mar 10;16:1530296. doi: 10.3389/fmicb.2025.1530296 (PMC11931161; doi:10.3389/fmicb.2025.1530296)
Supplement: Supplementary file 1 [file Data_Sheet_1.docx]

**The application of compost amended with biochar on the distribution of antibiotic resistance genes in a soil-cucumber system：From the perspective of high-dose fertilization**

Shuai Shi^a, b,^ ^†^, Zhenye Tong^a, c, †^, Bo Sun^a^, Yu Tian^a^, Qihui Zuo^a^, Xingxing Qiao^a^, Jiaze Duan^d^, Wenlong Bi^a^, Junmei Qin^a^, Jun Zhou^c^, Fenwu Liu^a, e, *^

^a^College of Resources and Environment, Shanxi Agricultural University, Jinzhong 030801, China;

^b^Shanxi Dadi Environment Investment Holdings Co., Ltd., Taiyuan 030000, China;

^c^Research Institute, College of Biotechnology and Pharmaceutical Engineering, Nanjing Tech University, Nanjing, Jiangsu 211816, China;

^d^Nongshengyuan Family Farm, Jinzhong 030801, China;

^e^Key Laboratory of Sustainable Dryland Agriculture (Co-construction by Ministry and Province), Ministry of Agriculture and Rural Affairs, Shanxi Agricultural University, Jinzhong 030801, China;

*** Corresponding author:**

**Dr. Fenwu Liu**

College of Resources and Environment,

Shanxi Agricultural University, Jinzhong,

Shanxi 030801, China

† These authors share first authorship.

Tel (Fax): +86-354-6288322

E-mail address: lfwlfw2008@163.com (F. Liu)

**Supplementary Materials and methods**

### S 1. Determination of cucumber quality indexes

Determination of total soluble sugars in cucumber by Fehling’s reagents method: (1) Extraction: cut fresh cucumber into small slices, weigh a certain amount (e.g., 2.0 g) into a 50 mL centrifugal tube, add 80% ethanol solution (20 mL), and place it on a boiling water bath to reflux the extraction for 30 minutes; (2) Filtration: filter the filtrate through a filter paper after cooling, and collect the filtrate. (3) Determination: take 1mL of filtrate, add it to 50 mL of water, add 2 mL of Feline A solution and 2 mL of Feline B solution, heat it to boiling, record the degree of color development, compare it with the standard sugar solution, and calculate the content of sugar.

Determination of soluble proteins in cucumber by Kaumas Brilliant Blue method: (1) Extraction: Grind the cucumber tissues into a homogenate with a mortar and a grinding rod, add phosphate buffer (pH 7.0) for extraction, centrifuge to remove the precipitate, and collect the supernatant. (2) Determination: Take 0.1 mL of the supernatant, add 1.0 mL of Kaomas Brilliant Blue G-250 reagent, oscillate and mix, and let it stand for 10-15 minutes, then measure the absorbance at 595 nm with a spectrophotometer, compare it with the standard protein solution, and calculate the protein content.

Determination of vitamin C in cucumber by 2,6-dichlorophenol indophenol titration method: (1) Extraction: Grind the cucumber tissue with a mortar and a grinding rod, add a small amount of oxalic acid solution (0.1 mol/L) to fully extract and volume to 25 mL; (2) Determination: Take 2 mL of the extraction solution, add 2,6-dichlorophenol indophenol (0.05%) to titrate until the color of the solution becomes colorless, record the volume of titrant consumed, and convert it to vitamin C. The amount of vitamin C was determined by the titration method.

Determination of nitrate in cucumber by UV spectrophotometry method: (1) Extraction: Weigh about 5 g of cucumber, put it in a 100mL volumetric flask, add 50 mL deionized water (or 0.01 mol/L dipotassium hydrogen phosphate solution), and leave it to extract for 1 hour. Extracted solution through the large pore neutral resin adsorption column to remove most of the organic matter and suspended solids, discard the colloidal material in the effluent, collect clean filtrate standby; (2) Determination: fill the achromatic column: 20 mL of zinc sulfate solution diluted to 200 mL with deionized water, and then added to a certain concentration of sodium hydroxide solution 20 mL, mix well. The solution is called achromatic solution. Pour 100 mL of the achromatic solution saturated with methanol into the achromatic column, discard the effluent, and record the amount of effluent before and after achromatization. 50 mL of filtrate was poured into an achromatic column containing 20 mL of achromatic solution and the effluent from the column was discarded. It was then poured into a 100 mL volumetric flask, 1 mL of hydrochloric acid solution was added, and the volume was fixed to 100mL.Measurement: The components were poured into two colorimetric tubes, one for the reagent blank and one for the sample measurement. The absorbance of the reagent blank and the sample were determined at 220 nm and 275 nm, respectively, and then the content of nitrate was calculated.

Determination of nitrite in cucumber by naphthalene ethylenediamine hydrochloride method: (1) Extraction: weigh about 5 g of cucumber in a 100 mL volumetric flask, add 50 mL of deionized water, leave to extract for 1 h, filter and collect the filtrate; (2) accurately pipette 25 mL of filtrate into a 100 mL volumetric flask; add 1 mL of p-aminobenzenesulphonic acid solution and mix; add 1 mL of naphthalene ethylenediamine hydrochloride solution and mix immediately; leave to stand for 15 min (protected from light), then pour into a clean After 15 minutes (protected from light), pour into a clean cuvette, add deionized water to the scale; use the reagent blank (25 mL deionized water + 1 mL p-aminobenzenesulfonic acid solution + 1 mL naphthalene ethylenediamine hydrochloride solution) as the reference, and read the absorbance of the samples at the wavelength of 538 nm.

### S 2. Determination of basic physical and chemical indicators of soil

Fresh soil samples were dried in an oven at 105°C for 24 h until constant weight, and the water content of the samples was calculated by the subtraction method. 2.5 g of sample was mixed with 25 ml of ultrapure water, shaken in a shaker for 1 h, and left to stand for 30 min, then the values of pH and electrical conductivity were determined by a pH meter (PHS-3C, Shanghai, China). Soil samples were cauterized in a muffle furnace at 550°C for 4 h, and then the organic matter content of the samples was calculated by the subtraction method. 20.00 g of fresh soil sample (to the nearest 0.01 g) was weighed into a 200 ml triangular flask, 100 ml of 2 M potassium chloride solution was added, shaken for 1 h in a shaker and left to stand, the supernatant was aspirated and used to determine ammonium nitrogen, nitrate nitrogen and nitrous nitrogen using a flow analyzer (AMS Alliance, France). Soil samples were decocted with concentrated sulfuric acid, and then the total nitrogen content of the decoction was determined by semi-micro Kjeldahl method. Total phosphorus and total potassium in soil samples were determined by NaOH melt-molybdenum antimony colorimetric and flame photometric methods, respectively. Available phosphorus, available potassium and available nitrogen in soil samples were determined by 0.5 mol/L NaHCO_3_ leaching- molybdenum antimony anti-coloring agent colorimetric method, 1 mol/L NH_4_OAC leaching-flame photometric method, and alkaline dissolution diffusion method.

**Table S1.** The usage of composts and chemical fertilizers in soils of different groups

| Fertilizer types | Different fertilization groups | | | |
| --- | --- | --- | --- | --- |
|  | CK group | TC group | LBTC group | SBTC group |
| Composts  (kg/each plot) | - | 11.24 | 11.24 | 11.24 |
| Urea (N≥46%)  (g/each plot) | - | 502.50 | 547.45 | 524.42 |
| Calcium superphosphate  (P_2_O_5_≥12%) (g/each plot) | - | - | 178.18 | 132.76 |
| Potassium sulfate  (K_2_O≥60%) (g/each plot) | - | 306.90 | 290.36 | 241.26 |

(-: The usage of the corresponding fertilizers was 0; CK group: soils without composts application; TC group: soils with traditional composts application; LBTC group: soils with larg size biochar-amended compost application; SBTC group: soils with small size biochar-amended compost application)

**Table S2.** The physico-chemical properties and the total absolute abundance of ARGs and MGEs in soils of different treatments before composts application ([Tong et al., 2023](#_ENREF_1))

| **Indicators** | | **Different groups** | | | |
| --- | --- | --- | --- | --- | --- |
|  |  | **CK group** | **TC group** | **LBTC group** | **SBTC group** |
| Physicochemical properties | Organic matter (g/kg) | 15.34 ± 0.15c | 16.32 ± 0.11b | 17.13 ± 0.41b | 18.71 ± 0.92a |
|  | AP (mg/kg) | 61.34 ± 1.96c | 96.71 ± 3.76b | 102.58 ± 1.24b | 113.86 ± 7.34a |
|  | AK (mg/kg) | 544.86 ± 7.71d | 609.26 ± 6.16c | 638.52 ± 2.96b | 671.99 ± 25.95a |
|  | NH_4_^+^-N (mg/kg) | 1.43 ± 0.10d | 6.15 ± 1.04a | 4.56 ± 0.97b | 2.96 ± 0.08c |
|  | NO_3_^-^-N (mg/kg) | 35.90 ± 0.60c | 42.97 ± 0.74b | 47.08 ± 0.89b | 56.15 ± 5.47a |
|  | pH | 8.72 ± 0.02a | 8.70 ± 0.05a | 8.69 ± 0.03a | 8.67 ± 0.01a |
| Genes | ARGs (copies/g) | 7.34×10^6^ ± 5.55×10^5^d | 6.94×10^7^ ± 8.01×10^6^a | 5.15×10^7^ ± 9.96×10^5^b | 4.18×10^7^ ± 8.29×10^5^c |
|  | MGEs (copies/g) | 4.83×10^4^ ± 3.09×10^3^d | 5.66×10^5^ ± 1.96×10^4^a | 5.00×10^5^ ± 3.16×10^4^b | 4.42×10^5^ ±1.00×10^4^c |

(CK group: soils without composts application; TC group: soils with traditional composts application; LBTC group: soils with large size biochar-amended compost application; SBTC group: soils with small size biochar-amended compost application)

**Table S3.** Relevant sequences of primer for the amplification of ARGs and MGEs

| Gene | F | R | |
| --- | --- | --- | --- |
| *16s* | GGGTTGCGCTCGTTGC | ATGGYTGTCGTCAGCTCGTG | |
| *tetA* | CTCACCAGCCTGACCTCGAT | CACGTTGTTATAGAAGCCGCATAG | |
| *tetB* | AGTGCGCTTTGGATGCTGTA | AGCCCCAGTAGCTCCTGTGA | |
| *tetC* | ACTGGTAAGGTAAACGCCATTGTC | ATGCATAAACCAGCCATTGAGTAAG | |
| *tetG* | TCAACCATTGCCGATTCGA | TGGCCCGGCAATCATG | |
| *tetM* | CATCATAGACACGCCAGGACATAT | CGCCATCTTTTGCAGAAATCA | |
| *tetO* | ATGTGGATACTACAACGCATGAGATT | TGCCTCCACATGATATTTTTCCT | |
| *tetQ* | CGCCTCAGAAGTAAGTTCATACACTAAG | TCGTTCATGCGGATATTATCAGAAT | |
| *tetW* | ATGAACATTCCCACCGTTATCTTT | ATATCGGCGGAGAGCTTATCC | |
| *tetX* | AAATTTGTTACCGACACGGAAGTT | CATAGCTGAAAAAATCCAGGACAGTT | |
| *tetZ* | CCTTCTCGACCAGGTCGG | ACCCACAGCGTGTCCGTC | |
| *sul1* | CACCGGAAACATCGCTGCA | AAGTTCCGCCGCAAGGCT | |
| *sul2* | GTCAAAGAACGCCGCAATGT | TCATCTGCCAAACTCGTCGTTA | |
| *gyrA* | CCAACAATGACCGACATCGC | GCGGTTAGATGAGCGACCTT | |
| *qnrS* | GTGAGTAATCGTATGTACTTTTGC | AAACACCTCGACTTAAGTCT | |
| *ermB* | TAAAGGGCATTTAACGACGAAA | TTTATACCTCTGTTTGTTAGGGAATTGAA | |
| *ermC* | TTTGAAATCGGCTCAGGAAAA | ATGGTCTATTTCAATGGCAGTTACG | |
| *ermF* | CAGCTTTGGTTGAACATTTACGAA | AAATTCCTAAAATCACAACCGACAA | |
| *ermT* | CATATAAATGAAATTTTGAG | ACGATTTGTATTTAGCAACC | |
| *mefA* | CCGTAGCATTGGAACAGCTTTT | AAACGGAGTATAAGAGTGCTGCAA | |
| *mphA* | CTGACGCGCTCCGTGTT | GGTGGTGCATGGCGATCT | |
| *intI1* | CGAACGAGTGGCGGAGGGTG | TACCCGAGAGCTTGGCACCCA |  |
| *intI2* | TGCTTTTCCCACCCTTACC | GACGGCTACCCTCTGTTATCTC |  |


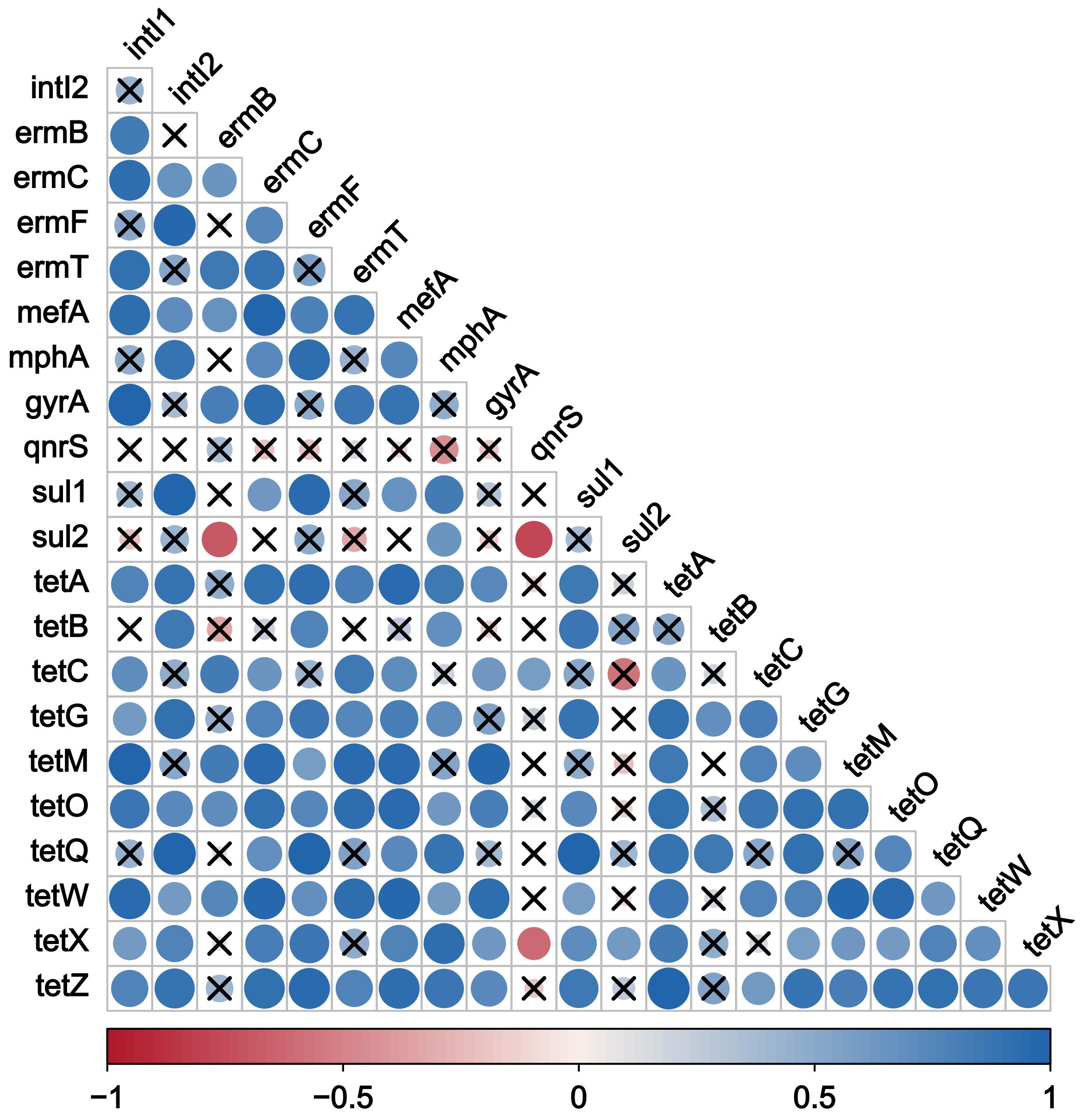


**Fig. S1.** The correlation between ARGs and MGEs in soils based on Person’s analysis (× represents insignificant correlation, *p > 0.05*)


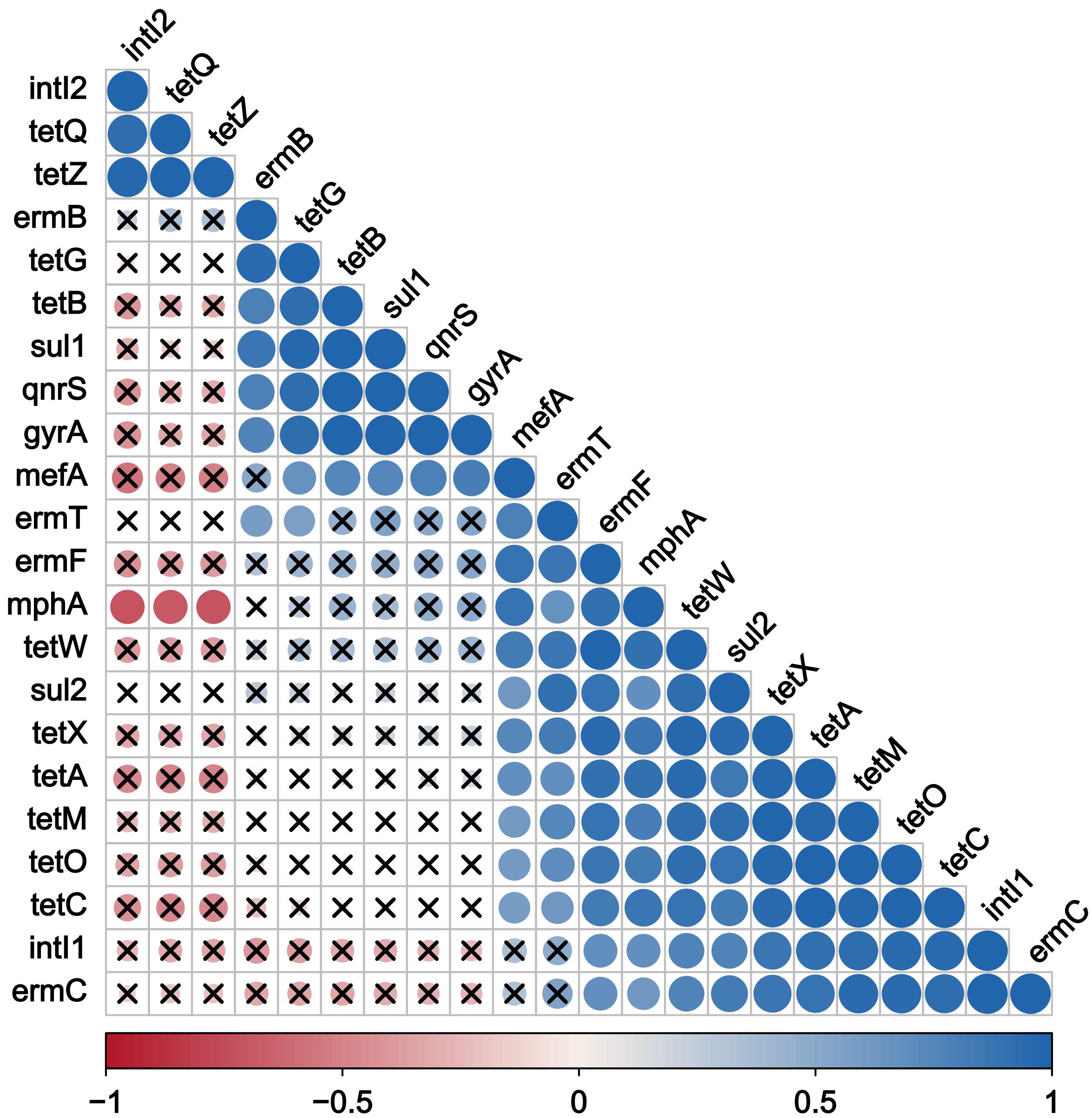


**Fig. S2.** The correlation between ARGs and MGEs in cucumbers based on Person’s analysis (**×** represents insignificant correlation, *p > 0.05*)

**
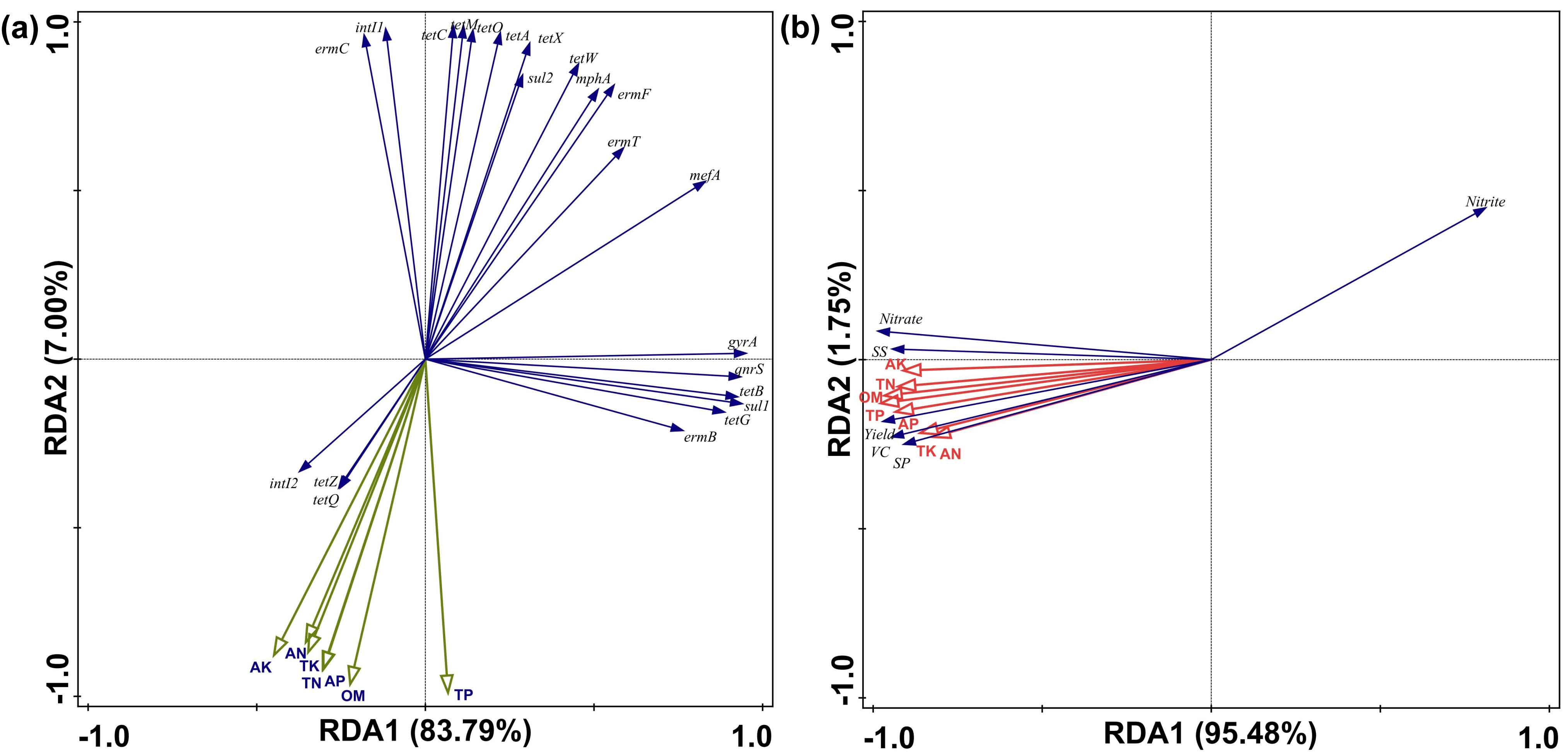
Fig. S3.** Contributions of nutrient indicators to ARGs and MGEs in cucumber (a) and to cucumber yield and quality indicators (b) were calculated based on Redundancy analysis (OM: organic matter; TN: total nitrogen; TP: total phosphorus; TK: total potassium; AN: available nitrogen; AP: available phosphorus; AK: Available potassium; SS: soluble total sugar; SP: soluble protein; VC: vitamin C)

**References:**

Tong, Z., Liu, F., Rajagopalan, U. M., Sun, B., Tian, Y., Zuo, Q., et al. Effect of biochar-containing compost on cucumber quality and antibiotic resistance genes abundance in soil-cucumber system. Sustainability (2023) 15, 9563. doi: <https://doi.org/10.3390/su15129563>
